# MYL2 as a potential predictive biomarker for rhabdomyosarcoma

**DOI:** 10.1097/MD.0000000000027101

**Published:** 2021-10-01

**Authors:** Junning Wang, Shang Gao, Keqin Dong, Peiyuan Guo, Meng-jie Shan

**Affiliations:** a The second Department of orthopedics, Hangzhou Fuyang District First People's Hospital, No. 429 Beihuan Road, Fuyang District, Hangzhou 311499, P.R. China; b Bethune Second Clinical Medical College of Jilin University, 218Ziqiang Hutong, Nanguan District, Changchun City, Jilin Province 130041, China; c School of Basic Medical Sciences, Hebei Medical University, 361 Zhongshan East Road, Shijiazhuang, Hebei 050017, P.R. China; d Graduate School, Chinese Academy of Medical Sciences & Peking Union Medical College, No. 9 Dongdansantiao, Dongcheng District, Beijing 100730, China; e Department of plastic surgery, Peking Union Medical College Hospital, Beijing, 100730, China.

**Keywords:** bioinformatics, biomarker, MYL2, prognosis, rhabdomyosarcoma

## Abstract

Rhabdomyosarcoma (RMS) is a common malignant soft tissue sarcoma, which is the third most common soft tissue sarcoma after malignant fibrohistoma and liposarcoma. The discovery of potential postbiomarkers could lead to early and more effective treatment measures to reduce the mortality of RMS. The discovery of biomarker is expected to be the direction of targeted therapy, providing a new direction for the precise treatment of RMS.

Gene Expression Omnibus database was used to download the tow gene profiles, GSE28511 and GSE135517. GEO2R was applied to identify differently expressed genes (DEGs) between RMS and normal group. Database for Annotation, Visualization and Integrated Discovery and Metascape can perform the enrichment analysis for the DEGs. Protein-protein interaction network was constructed, and the hub genes was identified by the Cytoscape. Expression and overall survival analysis of hub genes were performed.

A total of 15 common DEGs were screened between RMS and normal tissues. The enrichment analysis here showed that the DEGs mainly enriched in the muscle filament sliding, myofibril, protein complex, sarcomere, myosin complex, nuclear chromosome, and tight junction. The 6 hub genes (DNA Topoisomerase II Alpha, Insulin Like Growth Factor 2, HIST1H4C, Cardiomyopathy Associated 5, Myosin Light Chain 2 [MYL2], Myosin Heavy Chain 2) were identified. Compared with the normal tissues, MYL2 were down-regulated in the RMS tissues. RMS patients with low expression level of MYL2 had poorer overall survival times than those with high expression levels (*P* < .05).

In summary, lower expression of MYL2 was 1 prediction for poor prognosis of RMS. MYL2 is hope to be the target of therapy, which leads to more effective treatment and reduces the mortality rate of RMS.

## 1 Introduction

Rhabdomyosarcoma (RMS) is a malignant tumor arising from or derived from mesenchymal cells. RMS is a common malignant soft tissue sarcoma. It is most common in children and adolescents, and accounts for about 3% of all malignant tumors in children.^[1]^ RMS is the third most common soft tissue sarcoma after malignant fibrohistoma and liposarcoma. RMS has a high degree of malignancy and a poor prognosis.^[2]^ There is generally no typical radiological characteristics, and no calcification. Tumors can invade and destroy adjacent bones, especially the skull, forearm, hand, and foot. At present, the main clinical treatment means are radiotherapy and chemotherapy, and there are no clear target drugs.^[3]^ Radiation therapy and chemotherapy have a large side effect, so it is critical to look for targets to accurately treat RMS and improve patient survival. The cause of RMS is unknown. It is a soft tissue malignancy composed of rhabdomyocytes with different degrees of differentiation. The disease may be associated with genetic factors, chromosomal abnormalities, gene fusion, and other factors. Therefore, in-depth study of the molecular mechanism of RMS is particularly important.

Bioinformatics technology is the processing and analysis of all kinds of genome data, and it is also an important content of genome research. Bioinformatics is an indispensable part of genomics research.^[4]^ The development of bioinformatics is not only the analysis of genome data, but also the comprehensive analysis of known or new gene products. Bioinformatics is a science that used computers as tools to store, retrieve, and analyze biological information in the study of life sciences. It is one of the important frontier fields of life science and natural science, and will also be one of the core fields of natural science in the 21st century.^[5]^ The development of this technique has made it possible to explore and identify genetic targets related to diseases.^[6]^

Myosin Light Chain 2 (MYL2) is a protein-coding gene. Pathways associated with MYL2 include Guidance Cues and Growth Cone Motility and Activation of cAMP-Dependent proteinkinase A. The Gene Ontology annotation associated with the MYL2 includes calcium ion binding and actin monomer binding.^[7]^ However, the relationship between MYL2 and RMS is unclear.

Therefore, this paper intends to use bioinformatics technology to excavate the differentially expressed genes and hub genes between RMS and normal tissue, and conduct enrichment analysis, pathway analysis, and survival analysis. RMS is a significant harm to child injuries, and the discovery of targeted therapy is essential for treatment. The exploration of biomarkers related to the prognosis of RMS is expected to be the research direction of targeted therapy and provide a new direction for the precise treatment of RMS. In addition, as a biomarker of poor prognosis, MYL2 can also guide the timely selection of effective clinical management measures to reduce the mortality of RMS.

## 2 Material and methods

### 2.1 Dataset

We downloaded tow gene profiles, GSE28511 and GSE135517, from the Gene Expression Omnibus (GEO) database (https://www.ncbi.nlm.nih.gov/geo/). The GSE28511 includes 18 RMS samples and 6 normal samples, while the GSE135517 includes 48 RMS samples and 15 normal samples.

### 2.2 DEGs identification

We applied GEO2R (http://www.ncbi.nlm.nih.gov/geo/geo2r), an online tool based on GEOquery and limma R packages, to identify differently expressed genes (DEGs) between esophagus cancer and normal group. The cutoff criteria were that *P* value ≤.05 and a log (fold change) ≥1 or log (fold change) ≤–1.

### 2.3 DEGs annotation

Database for Annotation, Visualization and Integrated Discovery (DAVID) (https://david.ncifcrf.gov/home.jsp) and Metascape (http://metascape.org/gp/index.html) are 2 powerful annotation tools which can perform the biological process, cellular component, molecular function, and Kyoto Encyclopedia of Genes and Genomes analysis on genes. We annotated the function of common DEGs through DAVID and Metascape.

### 2.4 Protein-protein interaction network construction

The Search Tool for the Retrieval of Interacting Genes (http://string-db.org), can convert DEGs into expressed proteins and construct protein-protein interaction (PPI) network. We got PPI network of common DEGs through Search Tool for the Retrieval of Interacting Genes, and visualized it by Cytoscape (version 3.7.2).

### 2.5 Hub genes identification and expression

The cytoHubb was applied to screened out hub genes from the PPI network when the degree ≥6. Then, the clustering analysis of expression level of hub genes was performed using heatmaps based on the GSE28511 and GSE135517.

### 2.6 Overall survival analysis of hub genes

Effect of hub gene expression for overall survival was analyzed by the Kaplan-Meier Plotter (http://kmplot.com/analysis/index.php?p=background). The Kaplan Meier plotter is capable to assess the effect of 54k genes (mRNA, miRNA, protein) on survival in 21 cancer types. Primary purpose of the tool is a meta-analysis based discovery and validation of survival biomarkers. Furthermore, the Gene Expression Profiling Interactive Analysis (GEPIA, http://gepia.cancer-pku.cn/) was also used to analyze the role of hub genes on the overall survival so as to verify the results of Kaplan-Meier Plotter.

## 3 Results

### 3.1 DEGs

**Figure 1:**
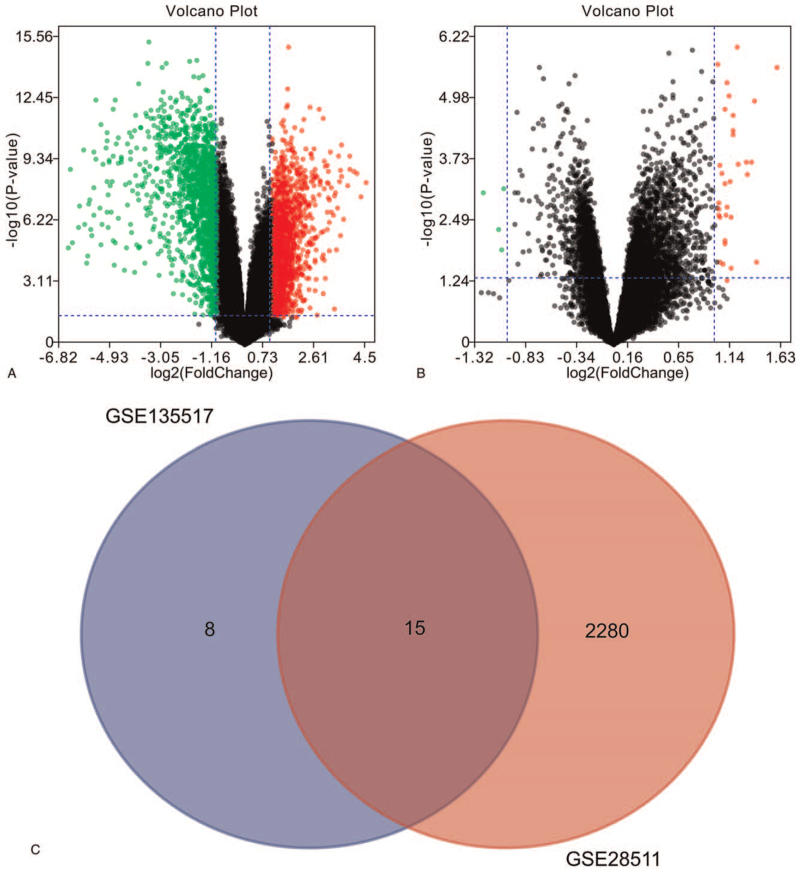
The DEGs. (A) The volcano map presenting DEGs in the GSE28511. (B) The volcano map presenting DEGs in the GSE135517. (C) The Venn diagram revealed 15 DEGs shared by the 2 datasets. DEG = differently expressed genes.

Two volcano plots present the DEGs in the GSE28511 (Fig. 1A) and GSE135517 (Fig. 1B). The Venn diagram revealed 15 DEGs shared by the 2 datasets (Fig. 1C).

### 3.2 Enrichment analysis of DEGs with DAVID

Through DAVID analysis, the results of the Gene Ontology analysis here showed that the DEGs mainly enriched in the muscle filament sliding, rhythmic process, myofibril, protein complex, a band, sarcomere, myosin complex, nuclear chromosome, and tight junction (Table 1).

**Table 1:** Functional enrichment of DEGs in rhabdomyosarcoma.

Term	Description	Count in gene set	*P* value
GO:0060047	Heart contraction	2	.008486
GO:0030049	Muscle filament sliding	2	.02462
GO:0048511	Rhythmic process	2	.034821
GO:0006936	Muscle contraction	2	.067921
GO:0044267	Cellular protein metabolic process	2	.074661
GO:0030016	Myofibril	3	2.05E-04
GO:0043234	Protein complex	4	.003468
GO:0031672	A band	2	.010705
GO:0030017	Sarcomere	2	.029558
GO:0016459	Myosin complex	2	.037747
GO:0000228	Nuclear chromosome	2	.039969
hsa04530	Tight junction	2	.049648

DEGs = differentially expressed genes, GO = Gene Ontology.

### 3.3 DEGs annotation with Metascape

**Figure 2:**
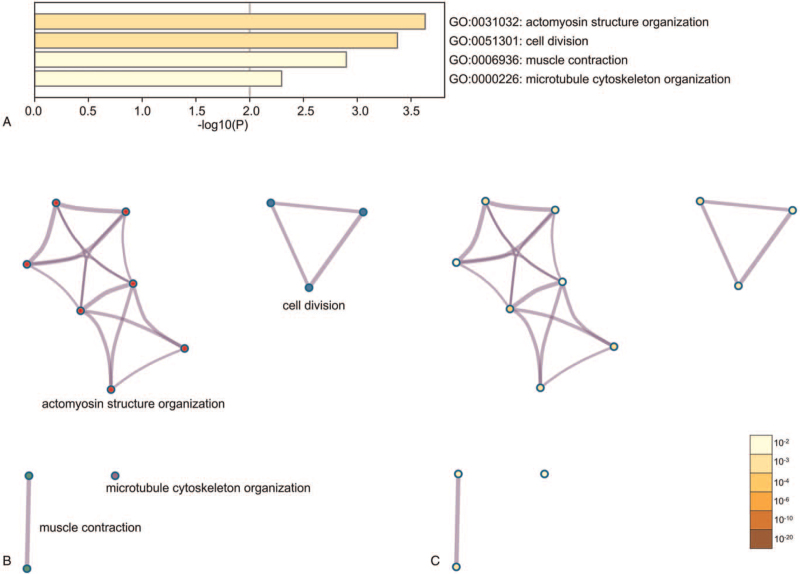
The enrichment analysis of DEGs by Metascape. (A) Heatmap of enriched terms across input differently expressed gene lists, colored by *P* values, via the Metascape. (B) Network of enriched terms colored by cluster identity, where nodes that share the same cluster identity are typically close to each other. (C) Network of enriched terms colored by *P* value, where terms containing more genes tend to have a more significant *P* value. DEG = differently expressed genes.

Enrichment analysis by Metascape was displayed in Figure 2. The DEGs related with biological process, cellular component, molecular function, and Kyoto Encyclopedia of Genes and Genomes enrichment analysis were displayed in actomyosin structure organization, cell division, muscle contraction, microtubule cytoskeleton organization (Fig. 2A, B, C).

### 3.4 Protein-protein interaction network and hub genes

**Figure 3:**
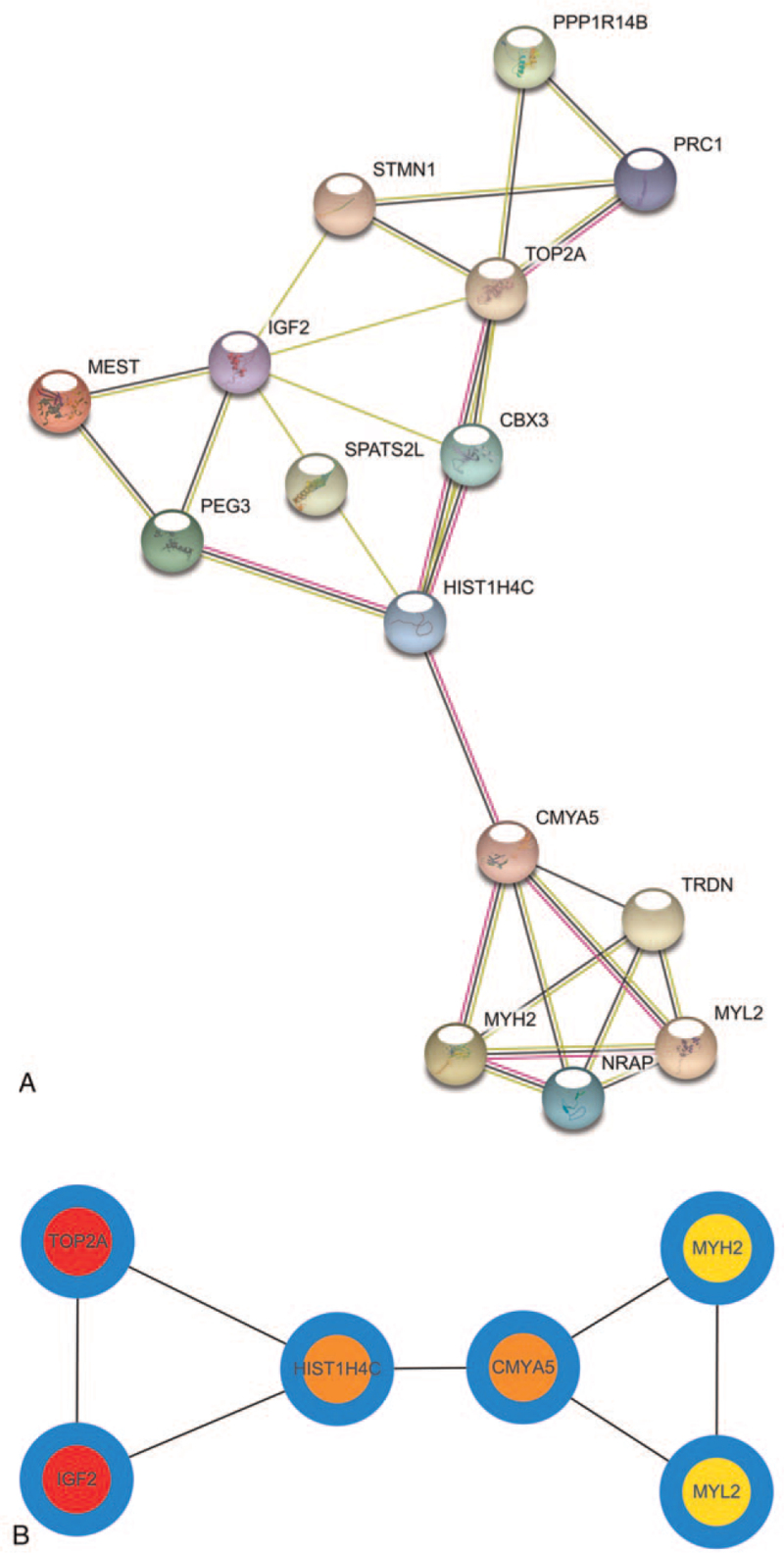
Protein-protein interaction network and hub genes. (A) PPI network showed that there exists closed relationship among DEGs. (B) The 6 hub genes (TOP2A, IGF2, HIST1H4C, CMYA5, MYL2, MYH2). CMYA5 = Cardiomyopathy Associated 5, DEG = differently expressed genes, IGF2 = Insulin Like Growth Factor 2, MYH2 = Myosin Heavy Chain 2, MYL2 = Myosin Light Chain 2, PPI = protein-protein interaction, TOP2A = DNA Topoisomerase II Alpha.

PPI was displayed in Figure 3A, which showed that there exists closed relationship among DEGs. The 6 hub genes (DNA Topoisomerase II Alpha [TOP2A], Insulin Like Growth Factor 2 [IGF2], HIST1H4C, Cardiomyopathy Associated 5 [CMYA5], MYL2, Myosin Heavy Chain 2 [MYH2]) were shown in Figure 3B.

### 3.5 The validation of expression level of hub genes

**Figure 4:**
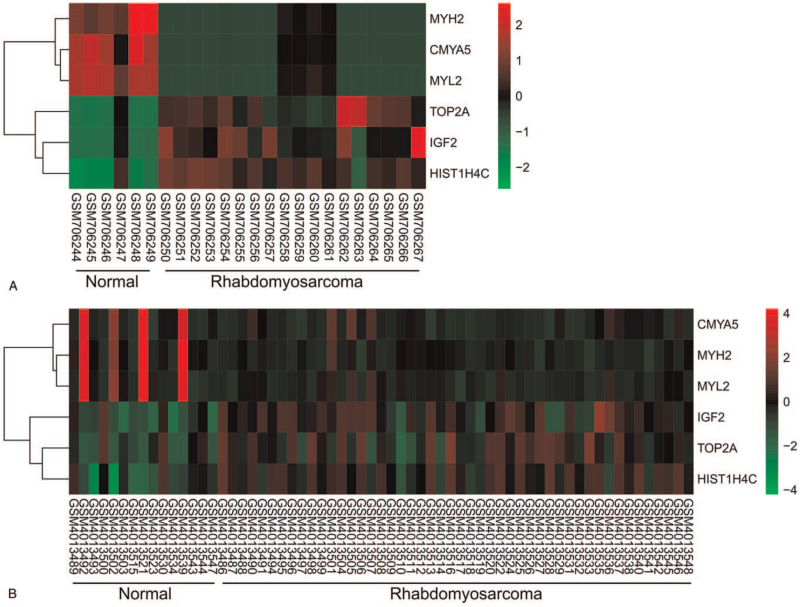
Two heat maps showed the expressions of the hub genes. (A) GSE28511; (B) GSE135517. CMYA5 = Cardiomyopathy Associated 5, IGF2 = Insulin Like Growth Factor 2, MYH2 = Myosin Heavy Chain 2, MYL2 = Myosin Light Chain 2, TOP2A = DNA Topoisomerase II Alpha.

Two heat maps showed the expressions of the hub genes in GSE28511 (Fig. 4A) and GSE135517 (Fig. 4B). Hierarchical clustering allowed for simple differentiation of RMS tissues from normal tissues via the expression levels of hub genes. Compared with the normal tissues, MYH2, CMYA5, and MYL2 were down-regulated in the RMS tissues. However, the expression of TOP2A, IGF2, and HIST1H4C in the RMS tissues was higher than the normal tissues.

### 3.6 Association between hub gene expression and overall survival via the Kaplan-Meier Plotter

**Figure 5:**
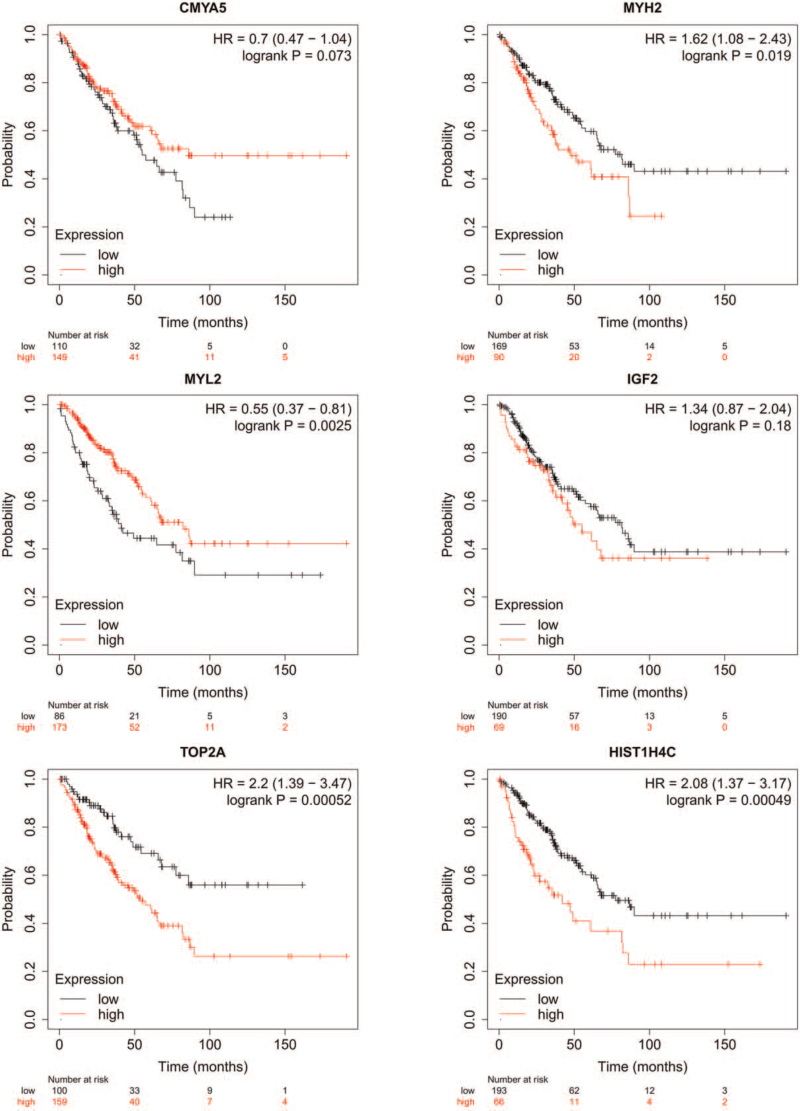
Association between hub gene expression and overall survival via the Kaplan-Meier Plotter. CMYA5 = Cardiomyopathy Associated 5, IGF2 = Insulin Like Growth Factor 2, MYH2 = Myosin Heavy Chain 2, MYL2 = Myosin Light Chain 2, TOP2A = DNA Topoisomerase II Alpha.

RMS patients with high expression levels of MYH2, TOP2A, and HIST1H4C had poorer overall survival times than those with low expression levels (*P* < .05; Fig. 5). RMS patients with low expression level of MYL2 had poorer overall survival times than those with high expression levels (*P* < .05; Fig. 5). However, there was no statistically significant effect on overall survival associated with the expression of CMYA5 and IGF2 (*P* > .05; Fig. 5).

### 3.7 Verification for the role of hub genes on the overall survival with the GEPIA

**Figure 6:**
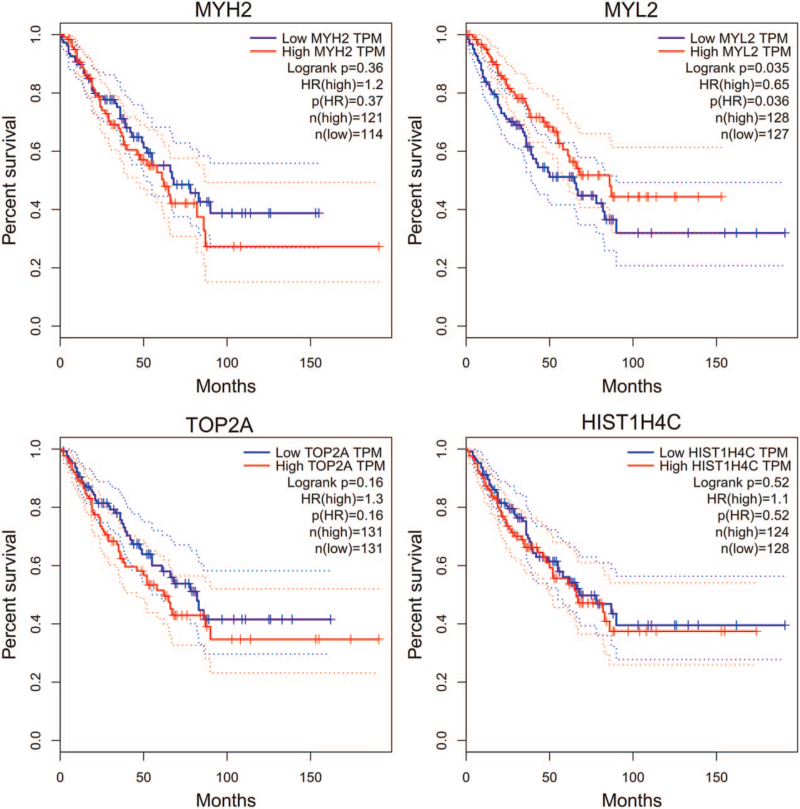
Verification for the role of hub genes on the overall survival with the GEPIA. GEPIA = Gene Expression Profiling Interactive Analysis, MYH2 = Myosin Heavy Chain 2, MYL2 = Myosin Light Chain 2, TOP2A = DNA Topoisomerase II Alpha.

The significant hub genes (MYH2, MYL2, TOP2A, and HIST1H4C) in the Kaplan-Meier Plotter were entered in the GEPIA. RMS patients with low expression level of MYL2 had poorer overall survival times than those with high expression levels (*P* < .05; Fig. 6). However, there was no statistically significant effect on overall survival associated with the expression of MYH2, TOP2A, and HIST1H4C (*P* > .05; Fig. 6).

## 4 Discussion

As a common malignant soft tissue tumor in children, RMS has high degree of malignancy, low survival rate, and unclear pathogenesis.^[1]^ In-depth exploration of the molecular mechanism of RMS is very important for the research of targeted drugs. In this study, bioinformatics techniques were used to analyze RMS and normal tissues to screen differentially expressed genes. The main result of this study was that the expression of MYL2 gene was lower in RMS than the normal tissues. Patients with RMS have a poor prognosis when MYL2 is poorly expressed.

MYL2 is a member of the myosin light chain family. As a regulatory light chain, MYL2 is a small molecule protein composed of 167 amino acids located on chromosome 17.^[8]^ Different types of muscle fibers contain different types of myosin light chain isomers. In vertebrate rhabdomyosin, the genes encoding the light chain subunits of rhabdomyosin include MYLPF, MYL7, and MYL2. Early studies found that MYL2 gene is mainly concentrated in muscle tissues, and the mutation of this gene can lead to muscle R cell proliferation. Earlier studies found that MYL2 gene is mainly concentrated in muscle tissue, and the mutation of this gene can lead to muscle cell proliferation.^[9]^ In patients with muscle-related diseases, the expression of MYL2 gene is significantly lower than that of normal people, while the expression of MYL2 gene is significantly lower in patients with severe diseases than that in patients with mild diseases.^[10]^ Recent studies have shown that phosphorylation of MYL2 in adult muscle cells causes muscle dilation,^[11]^ which was related with numerous muscle-related diseases.^[12]^ This suggests that MYL2 plays an important role in the normal maintenance of muscle cells. One study has shown that MYL2 gene is expressed in mature mouse muscle tissue but not in the differentiation of C2C12 myoblasts cultured in vitro.^[7]^ When the gene was knocked out of the muscle cells of mice, their embryonic or primary growth was severely abnormal.^[13]^ In fibroblasts, the integrity of muscle fiber type mainly be maintained by MYL2 genes. MYL2 plays a role of regulating the activity of muscle fibers, transformation in the muscle fiber type, and affects the growth of muscle.^[14]^ Therefore, it is speculated that MYL2 gene plays an important role in muscle growth and development, and may be a candidate gene for regulating muscle development.

One study has shown that MYL2 promoter is a muscle-specific promoter, and multiple binding sites that can promote the proliferation and differentiation of muscle cells are predicted in the upstream of the initial codon.^[15]^ And MYL2 gene is speculated to be involved in the regulation of the growth and differentiation of skeletal muscle cells. The researchers cloned the MYL2 gene, a promoter specifically expressed in muscle.^[16]^ The muscle specificity of the promoter was determined by dual-luciferase assay, and it was found that the MYLP2 promoter contained the MEF2 binding site near the starting site, which could activate the expression of the chloramphenicol acetyltransferase reporter gene, and played a very important role in zebrafish muscle cells.^[17]^ In addition, 1 study has shown that goat MYL2 protein is specifically expressed in goat skeletal muscle fibers, mainly in type I muscle fibers (slow contraction muscle fibers).^[18]^ MYL2 gene is closely related to the growth and development of muscle and plays a crucial role in the early growth and differentiation of muscle.^[7]^

MYL2 gene, as a member of the myosin light chain family, is a regulatory light chain. Early study found that the fundamental light chain plays an important role in the maintenance of the heavy chain configuration, while the regulatory light chain plays a regulatory role in the activity of muscle fibers.^[19]^ Therefore, it is believed that the myosin light chain plays an important role in the type and growth of muscle fibers. MYL2 is a regulatory light chain that regulates muscle fiber activity.^[19]^ Then it affects the activity of ATPase on the heavy chain of myosin and transforms the types of muscle fiber, and regulates the growth of muscle.^[20,21]^ MYL2 may play an important role in mammalian muscle growth and development. MYL2 gene was found to be expressed in skeletal muscle of mature mice, but not in the differentiation of myoblasts in vitro MYL20.^[13]^ When the MYL2 gene was knocked out of the heart muscle and skeletal muscle, the mice developed severe abnormalities in embryonic or primary growth.^[14,22]^ MYL2 plays an important role in the maintenance of muscle fiber integrity, cell structure, cell adhesion, cell migration, and cell division.^[23]^ Obtaining the length cDNA sequence of this gene is of great significance for further study of gene function.

The above literature review is consistent with our results. MYL2 gene expression is reduced in muscle-related diseases. Similarly, MYL2 gene is low expressed in RMS, and the normal development and growth of muscles are restricted at this time, and the proliferation and differentiation of muscle cells are also affected accordingly, eventually leading to the occurrence of tumors.

RMS is a significant harm to child injuries, and the discovery of targeted therapy is essential for treatment. The exploration of biomarkers related to the prognosis of RMS is expected to be the research direction of targeted therapy and provide a new direction for the precise treatment of RMS. In addition, as a biomarker of poor prognosis, MYL2 can also guide the timely selection of effective clinical management measures to reduce the mortality of RMS.

## 5 Limitation

Despite the rigorous bioinformatics analysis in this paper, there are still some deficiencies. In this study, MYL2 excavated by bioinformatics have not been verified at the cell and animal levels. Overexpression or knockout of MYL2 were conducted to further verify its function.^[24]^ Therefore, in the future research, we should carry out in-depth exploration in this aspect.

## 6 Conclusions

In summary, lower expression of MYL2 was 1 prediction for the poor prognosis of patients with RMS. The exploration of MYL2 related to the prognosis of RMS is expected to be the research direction of targeted therapy and provide a new direction for the precise treatment of RMS. In addition, as a biomarker of poor prognosis, MYL2 may also guide the timely selection of effective clinical management measures to reduce the mortality of RMS. MYL2 might provide novel research idea for the diagnosis and treatment of RMS.

## Author contributions

**Conceptualization:** Meng-jie Shan.

**Formal analysis:** Junning Wang, Shang Gao

**Investigation:** Junning Wang, Shang Gao

**Methodology:** Junning Wang, Meng-jie Shan.

**Project administration:** Peiyuan Guo.

**Software:** Keqin Dong.

**Supervision:** Shang Gao.

**Validation:** Keqin Dong, Peiyuan Guo.

**Visualization:** Peiyuan Guo.

**Writing – original draft:** Junning Wang.

**Writing – review & editing:** Shang Gao, Meng-jie Shan.
